# Considering adaptive genetic variation in climate change vulnerability assessment reduces species range loss projections

**DOI:** 10.1073/pnas.1820663116

**Published:** 2019-05-06

**Authors:** Orly Razgour, Brenna Forester, John B. Taggart, Michaël Bekaert, Javier Juste, Carlos Ibáñez, Sébastien J. Puechmaille, Roberto Novella-Fernandez, Antton Alberdi, Stéphanie Manel

**Affiliations:** ^a^Biological Sciences, University of Southampton, Southampton SO17 1BJ, United Kingdom;; ^b^School of Biological Sciences, University of Bristol, Bristol BS8 1TQ, United Kingdom;; ^c^Department of Biology, Colorado State University, Fort Collins, CO 80523-1878;; ^d^Institute of Aquaculture, University of Stirling, Stirling FK9 4LA, United Kingdom;; ^e^Estación Biológica de Doñana, Consejo Superior de Investigaciones Cientifica (CSIC), 41092 Seville, Spain;; ^f^Institut des Sciences de l’Evolution de Montpellier (ISEM), University of Montpellier, 34095 Montpellier, France;; ^g^Groupe Chiroptères de Midi-Pyrénées (GCMP), 31076 Toulouse, France;; ^h^Zoological Institute and Museum, University of Greifswald, 17489 Greifswald, Germany;; ^i^Evolutionary Genomics, University of Copenhagen, 1350 Copenhagen, Denmark;; ^j^Centre d’Écologie Fonctionnelle et Évolutive (CEFE), Université de Recherche Paris Sciences et Lettres (PSL), École Pratique des Hautes Études (EPHE), Université de Montpellier, 34293 Montpellier, France

**Keywords:** global climate change, genetic adaptations, ecological niche models, conservation genomics, evolutionary rescue

## Abstract

Local adaptations can determine the potential of populations to respond to environmental changes, yet adaptive genetic variation is commonly ignored in models forecasting species vulnerability and biogeographical shifts under future climate change. Here we integrate genomic and ecological modeling approaches to identify genetic adaptations associated with climate in two cryptic forest bats. We then incorporate this information directly into forecasts of range changes under future climate change and assessment of population persistence through the spread of climate-adaptive genetic variation (evolutionary rescue potential). Considering climate-adaptive potential reduced range loss projections, suggesting that failure to account for intraspecific variability can result in overestimation of future losses. On the other hand, range overlap between species was projected to increase, indicating that interspecific competition is likely to play an important role in limiting species’ future ranges. We show that although evolutionary rescue is possible, it depends on a population’s adaptive capacity and connectivity. Hence, we stress the importance of incorporating genomic data and landscape connectivity in climate change vulnerability assessments and conservation management.

## Significance

Forecasts of species vulnerability and extinction risk under future climate change commonly ignore local adaptations despite their importance for determining the potential of populations to respond to future changes. We present an approach to assess the impacts of global climate change on biodiversity that takes into account adaptive genetic variation and evolutionary potential. We show that considering local climatic adaptations reduces range loss projections but increases the potential for competition between species. Our findings suggest that failure to account for within-species variability can result in overestimation of future biodiversity losses. Therefore, it is important to identify the climate-adaptive potential of populations and to increase landscape connectivity between populations to enable the spread of adaptive genetic variation.

Climate change is predicted to result in widespread population and species extinctions (1), and climate-related local extinctions have already been observed in hundreds of species (2). However, an equivalent number of species did not experience local extinctions at their warm range edge (2), indicating that either phenotypic plasticity or genetic adaptations may enable some populations to persist under warmer conditions. This highlights the importance of incorporating intraspecific adaptations into climate change vulnerability assessments (3, 4). However, methodologies to adequately incorporate genomic data into projections of species responses to current and changing climatic conditions (5) and into conservation management strategies (6) are still missing.

Vulnerability to climate change is most commonly assessed based on forecasted distributional changes using ecological niche modeling approaches (also known as species distribution models), which project future changes in the distribution of suitable climatic conditions that characterize species’ current ranges (7). A major limitation of these approaches, which can lead to erroneous predictions and misplaced conservation efforts, is the disregard of intraspecific climatic adaptations and the consequent differences in population responses to climate change (8). Evidence of contrasting patterns of physiological variation in thermal tolerance among and within species highlights the importance of incorporating intraspecific variation in climatic adaptations into ecological niche models (ENMs) (9). However, such model improvements are limited by the paucity of observational and experimental studies of local climatic adaptations (10).

To date, studies attempting to incorporate genetic variation into ENMs primarily use neutral markers to identify phylogeographic structure and generate separate models for each genetically distinct population. These have resulted in more pessimistic forecasts than traditional ENMs, predicting increased threats from climate change due to range losses in vulnerable populations (11), but have not affected projections of range size changes at the species level (12). These attempts are limited in scope because neutral markers provide information on the species’ evolutionary history and barriers to gene flow but not on the ability of individuals to adapt and survive under changing conditions. Moreover, range shifts under future climate change are predicted to result in genetic homogenization across species ranges and loss of historic and current population subdivisions (13). More recent studies integrated genomic adaptations with ENM projections to identify vulnerable populations that will need to adapt to survive under future climate change (14, 15). However, genetic data related to intraspecific variation in climatic adaptations have yet to be directly incorporated into ENMs.

**Fig. 1.**
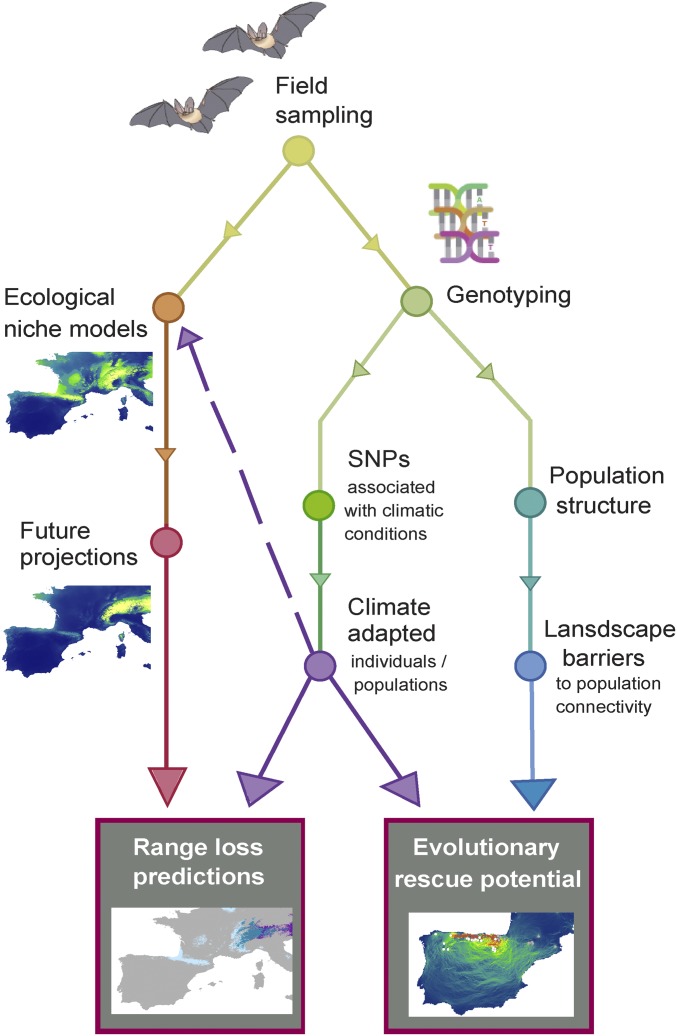
Framework for incorporating within-species climatic adaptations into projections of future range losses and evolutionary rescue potential.

To address this gap, our study develops an approach to forecast range changes under future climate change for individuals adapted to different climatic conditions and to determine the evolutionary rescue potential of populations [the ability of populations to persist through adaptation to the novel conditions (16)]. This requires first identifying local climatic adaptations in wild populations by using genotype–environment association (GEA) analysis and then incorporating this information directly into ENMs and projections of future range losses (Fig. 1). The applicability of our approach is tested using spatial and genomic data from a pair of cryptic Mediterranean bat species with relatively limited long-distance dispersal abilities, *Myotis escalerai* and *Myotis crypticus*, that have only recently been confirmed as separate species (17, 18). *M. escalerai* is endemic to the Iberian Peninsula (Spain, Portugal, and the Balearic Islands) and the eastern French Pyrenees, while *M. crypticus* is found across Italy, southern France, the Pyrenees, and the north of Spain (19). The current distributions of both species overlap across the north of the Iberian Peninsula but are likely limited by interspecific competition (20). As such, these species offer a good study system to simultaneously look at the effect of local climatic adaptations and interspecific interactions on the current and future distributions of species that are restricted to areas particularly vulnerable to the effects of climate change.

## Results and Discussion

### Incorporating Local Adaptations into Climate Change Vulnerability Projections.

Traditionally, local adaptations were identified through common garden experiments (3), but the advent of high-throughput sequencing techniques opened the door to the use of genomic approaches to identify signatures of local adaptations by relating genetic variation and environmental variables (21). We combined two GEA methods and used two key climatic variables that are likely to directly affect bat survival and reproductive success (maximum temperatures and summer rainfall; *SI Appendix*) to identify 32 potential climate-adaptive SNPs in *M. escalerai* and 38 in *M. crypticus* (see *SI Appendix* for population structure and GEA results; Dataset S1). It is important to note that our study is based on reduced-representation genomic datasets, which do not capture all adaptive genomic variation and therefore only offer an indication of SNPs under (or linked to) climate-related selection (22). However, subsetting our data illustrates how downstream results are robust to smaller numbers of SNPs (*SI Appendix*, Table S1).

Plotting the multilocus adaptive genotypes of individuals in a constrained ordination space, we classified 34% of *M. escalerai* individuals as adapted to hot–dry conditions, 50% as adapted to cold–wet conditions, and the rest as intermediate genotypes. Based on the proportion of these individuals in each population (sampled from cave roosts), we classified six *M. escalerai* populations, mainly from Portugal and southern Spain, as primarily adapted to hot–dry conditions; eight, mainly from northern Spain and the Pyrenees, as cold–wet adapted; and four as mixed (*SI Appendix*, Fig. S1). In *M. crypticus*, 45.6% of individuals were classified as adapted to hot–dry conditions, and 36.8% were classified as adapted to cold–wet conditions. Most of the cold–wet-adapted individuals were found in the Pyrenees, Alps, and Massif Central, France (*SI Appendix*, Fig. S2). Population data are not available for *M. crypticus* because it primarily roosts in trees and switches roosts regularly, and therefore, colony roost locations are unknown.

Intraspecific variation in local climatic adaptations was incorporated into ENMs by generating separate models for hot–dry- and cold–wet-adapted individuals and comparing predictions to models generated using all of the known geographic location records of each species. ENM projections are sensitive to variability resulting from the modeling approach, general circulation model (GCM), and greenhouse gas emission scenario used. To address these sources of variability, which can affect future range loss projections (23), we employ an ensemble modeling approach (24), averaging projection results across model algorithms, three GCMs, and two greenhouse gas emission scenarios representing the worst-case and a more moderate emissions scenario. All ENMs had strong support and good discrimination ability [mean values are true skills statistics (TSS): 0.766 ±0.03; area under the curve (AUC): 0.929 ±0.02; AUC cross validation: 0.866 ±0.03; Table 1 and *SI Appendix*, Table S2] and performed significantly better than random (null model AUC range: *M. escalerai* = 0.603–0.685; *M. crypticus* = 0.623–0.713). In both species, intraspecific overlap in ecological space (niche overlap) between cold–wet- and hot–dry-adapted individuals (*M. escalerai*: Schoener’s D = 0.432; *M. crypticus*: D = 0.465) is slightly lower than overlap between species (D = 0.480), although both are significantly lower than random (*SI Appendix*, Table S3). Our findings that levels of niche overlap were lower within than between species highlight the importance of incorporating intraspecific variation in climatic adaptations into ENM projections of species range shifts under climate change (8, 9).

**Table 1.** Results of the ecological niche models, including percentage of Iberia predicted to be climatically suitable under present and future (2070, RCP 8.5) conditions and percentage range changes within Iberia

Taxon	*n*	AUC ROC	TSS^a^	AUC-test	Percentage suitable, present	Percentage suitable, future	Percentage range change
*Myotis escalerai,* all	313	0.941	0.781	0.850	38.40	20.38	−46.94
*M. escalerai,* hot–dry	19	0.914	0.727	0.876	46.82	49.50	**+5.72**
*M. escalerai,* cold–wet	41	0.946	0.806	0.841	29.27	12.08	−58.73
*Myotis crypticus,* all	168	0.926	0.729	0.896	20.51	2.61	−87.28
*M. crypticus,* hot–dry	25	0.908	0.752	0.836	14.34	7.54	−47.42
*M. crypticus,* cold–wet	18	0.940	0.798	0.896	4.89	<0.01	−99.96

See *SI Appendix*, Table S2, for range change projections across the study area and for the RCP 4.5 scenario. AUC ROC, area under the receiver operating characteristic curve for ensemble models; AUC-test, AUC cross-validation scores for Maxent models; *n*, sample size. Bold denoted projected range increases.

^a^ For ensemble models.

**Fig. 2.**
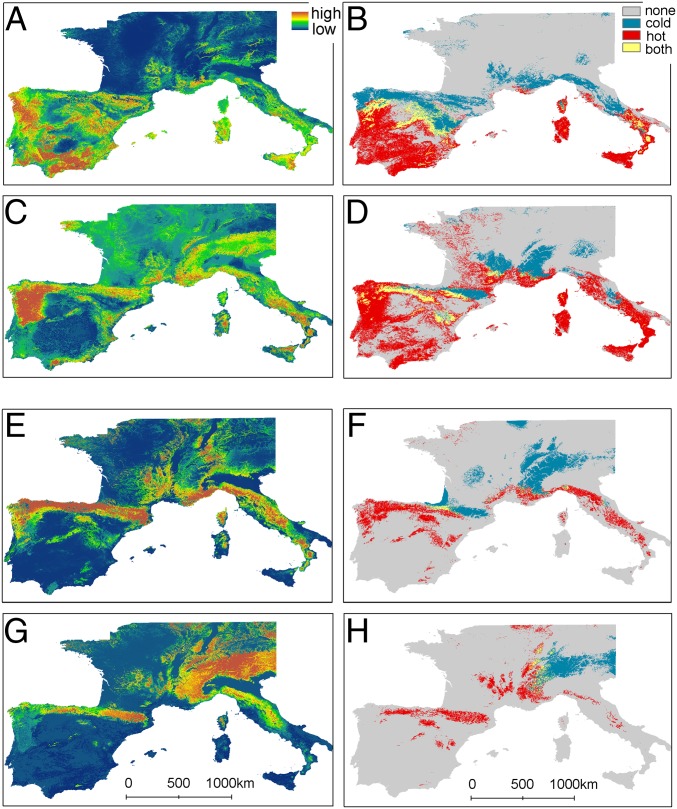
The effect of integrating intraspecific climatic adaptations into ecological niche model projections. Ecological niche modeling outputs for *M. escalerai* (*A*–*D*) and *M. crypticus* (*E*–*H*), including the full dataset (*A*, *C*, *E*, and *G*) and overlap between separate models for individuals adapted to hot–dry (red) and cold–wet (blue) conditions (*B*, *D*, *F*, and *H*) under present (*A*, *B*, *E*, and *F*) and future (2070, RCP 8.5) (*C*, *D*, *G*, and *H*) climatic conditions. Relative probability of occurrence ranges from low in blue to high in orange.

Considering local climatic adaptations in ENMs reduced future range loss projections. Based on the full dataset and worst-case scenario [Representative Concentration Pathway (RCP) 8.5 W/m^2^], *M. escalerai* is projected to lose 47% (range based on different GCMs is 38–53%) of its Iberian range by the end of this century but only 19% (range of 13–25%) based on the combined ranges of hot–dry- and cold–wet-adapted individuals, resulting in up to 60% reduction in projected Iberian range losses (16% based on the moderate scenario RCP 4.5 W/m^2^). Similarly, *M. crypticus* is projected to lose 87% (range of 75–94%) of its Iberian range based on the full dataset but only 58% (range of 44–68%) based on the combined adaptive ranges (33% reduction in projected losses with RCP 8.5 versus 40% reduction with RCP 4.5; Fig. 2 and *SI Appendix*, Fig. S3 for RCP 4.5; Table 1; *SI Appendix*, Table S2). There is a mismatch between the low extinction rates observed during Pleistocene climatic changes and the high rates forecasted by traditional future ENMs (25). Our findings suggest that incorporating adaptive intraspecific genetic variation is essential for realistic projections of species range losses under climate change and for preventing overestimation of future biodiversity losses.

Hot–dry-adapted *M. escalerai* individuals are the only group predicted to have substantial increases in climatic suitability across Europe (+34%) under future climate change. However, increases are projected mainly outside Iberia (Fig. 2 *B* and *D*), where the species is not currently found [except in the Pyrénées-Orientales in France (26)] and where it is likely to encounter interspecific competition with two cryptic congeners, *M. crypticus* and *Myotis nattereri sensu stricto* (19). However, more modest range gains (+5.7%) are also projected within Iberia. *M. escalerai* is restricted to its glacial refugia, likely due to range expansion limitations imposed by interspecific competition (20). The life history traits, habitat specialization, and restricted distribution of *M. escalerai* suggest it is particularly vulnerable to climate change (7). However, our study predicts that *M. escalerai* will be able to survive in situ across much of its currently occupied range as a result of its adaptive capacity.

Overall, hot–dry genotypes are predicted to expand their range at the expense of cold–wet genotypes. However, survival at the trailing (equatorward) edge of species ranges depends on the maximum thermal tolerance of the species. Species living in warm environments may be unable to physiologically adapt to increased heat because their niches are close to their upper thermal limits, which were shown to be phylogenetically conserved and therefore less likely to evolve (27). On the other hand, at least for ectotherms, the equatorward range limit does not reflect maximum warm temperature tolerance, and therefore, species may be able to physiologically tolerate higher thermal stress at their warm range limits under future climate change (28). Genomic data support the genetic basis of greater thermal tolerance in individuals living in warmer microclimates under higher heat stress (29). In contrast, individuals adapted to cold–wet conditions will experience the most severe range losses. Cold–wet *M. escalerai* genotypes are projected to lose more than half of their Iberian range and retract to mountain ranges (Fig. 2*D*), while cold–wet *M. crypticus* genotypes are projected to entirely disappear from Iberia and Italy with the exception of the Alps (Fig. 2*H* and Table 1). Bay et al. (4) show that populations exhibiting a strong mismatch between current local genetic adaptations and future climatic conditions have a higher likelihood of declining.

Considering adaptive variation increased the predicted potential for interspecific competition, through increased range overlap. Range overlap between species in Iberia was predicted to decrease under future conditions (84% reduction, from 10.5 to 1.7% of Iberia), but estimations of future range overlap were more than 4 times higher when the ranges of hot–dry and cold–wet individuals were combined (7.1%; *SI Appendix*, Fig. S3). Changing species interactions have already been implicated in population declines and extinctions related to climate change (30). Moreover, spatially explicit simulations of multispecies responses to climate change show that when interspecific competition is included in future models, preadapted species displace maladapted species (31), which is likely to be the outcome of increased future range overlap among the warm-adapted *M. escalerai* and the more cold-adapted *M. crypticus*.

Our results are supported by previous studies that used common garden experiments to show that incorporating information on local adaptations decreases future range loss projections for pines (32). Similarly, Bush et al. (33) showed that incorporating physiological measurements in hybrid ENMs that account for intensity of selection, response to selection, and dispersal probability reduces future range loss projections for *Drosophila*. Genomic studies of local adaptations offer an alternative approach to understanding adaptive responses to climate change when reciprocal transplant or common garden experiments are unfeasible due to biological, practical, or ethical reasons, as is the case with many vertebrates and species of conservation concern (3).

### Evolutionary Rescue Potential Is Limited by Landscape Connectivity.

We use gene flow as a result of the movement of adapted individuals between populations to estimate the ability of a population to avoid extinction due to environmental stress through adaptation to the changed environment (evolutionary rescue). Increased thermal tolerance can evolve over a few decades in small organisms with short generation time (34). However, in long-lived organisms with small population sizes, the potential for evolutionary rescue depends primarily on standing genetic variation and is facilitated in structured populations by local dispersal (16). Detecting local adaptations can help with identifying populations that will need evolutionary rescue, as well as potential donor populations that already show a signature of adaptations to warmer and drier conditions.

**Fig. 3.**
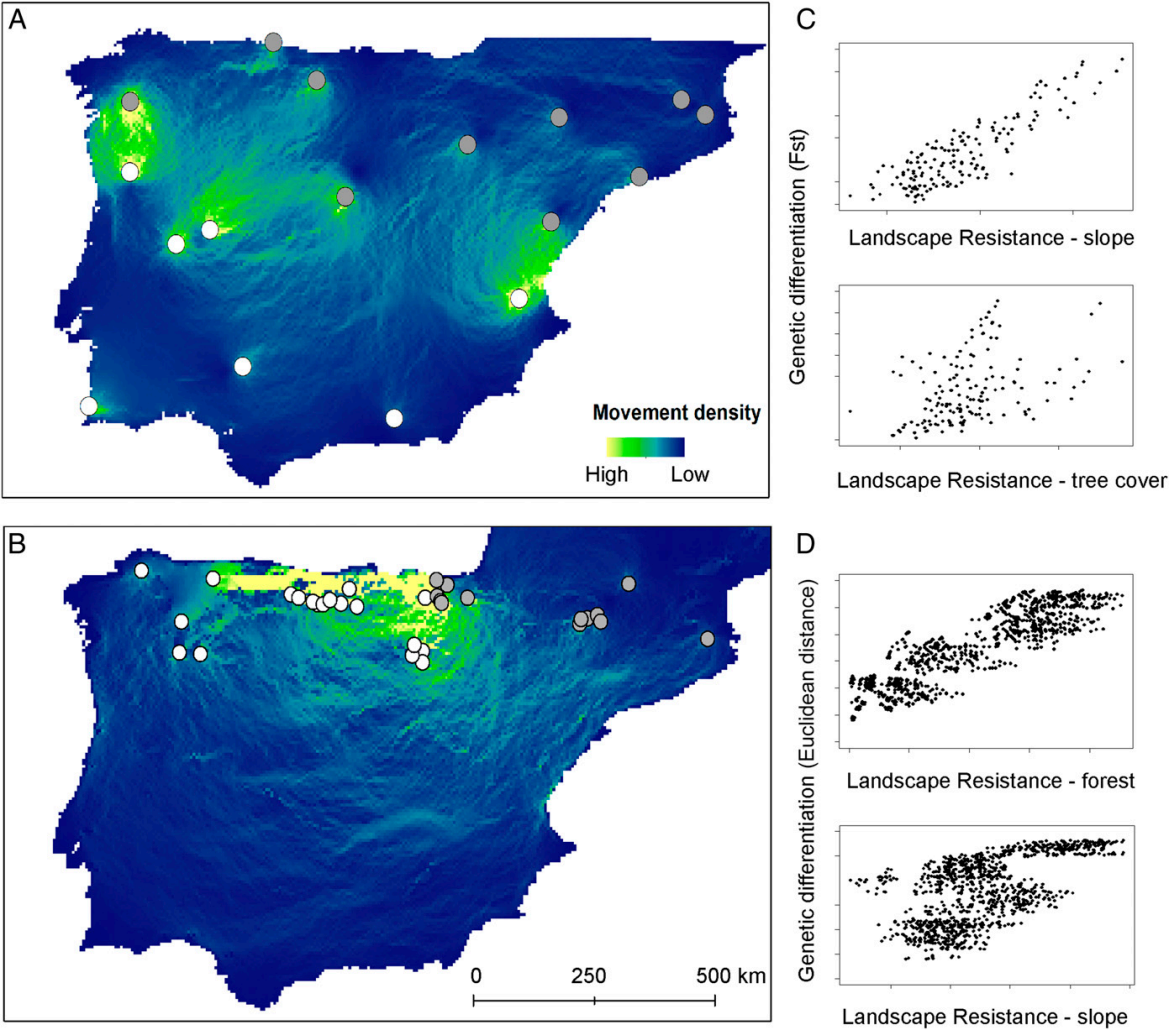
Modeling evolutionary rescue potential under future climate change in (*A*) *M. escalerai* and (*B*) *M. crypticus*, depicted as the predicted density of movement (yellow is high and blue is low) from populations or individuals adapted to hot–dry conditions (white circles) to those adapted to cold–wet conditions (gray circles) based on the effect of slope and tree cover (*A*) or slope and forest cover (*B*) on movement. The relationship between landscape resistance and genetic differentiation in *M. escalerai* (*C*) and *M. crypticus* (*D*). Fst is Wright’s fixation index measure of population differentiation resulting from genetic structure.

Given that the studied bat species are forest specialists, both range shifts and the movement of adaptive genetic variation among populations via individuals’ dispersal are likely to be limited by landscape connectivity. We use a landscape genetics approach (35) to first identify landscape barriers to gene flow and then extrapolate how these will affect the potential for evolutionary rescue from hot–dry- to cold–wet-adapted locations. Genetic connectivity in both species was most strongly related to the combination of forest cover and slope (*M. escalerai: R*^2^ = 0.532; *M. crypticus: R*^2^ = 0.356; see Fig. 3 *C* and *D* and *SI Appendix* for landscape genetics results; *SI Appendix*, Tables S4 and S5). Extrapolating these relationships to estimate gene flow potential from hot–dry- to cold–wet-adapted locations shows that landscape barriers to movement are likely to limit the ability of individuals adapted to hot–dry conditions to reach areas that will become climatically unsuitable for cold–wet-adapted individuals to prevent their extirpation under future climate change, even though these areas will become suitable for hot–dry genotypes (e.g., see Fig. 3*B* for *M. crypticus*). On the other hand, in *M. escalerai*, although hot–dry-adapted individuals are not likely to be able to reach areas like the eastern Pyrenees (Fig. 3*A*), future ENMs show that much of this area will remain climatically suitable for cold–wet-adapted individuals, suggesting that evolutionary rescue will not be necessary. However, it is important to note that gene flow in these forest bats is limited by forest cover, which is likely to change substantially under future climate change (36). Our data also reveal cold–wet locations that harbor individuals adapted to hot–dry conditions (and vice versa; *SI Appendix*, Figs. S1 and S2). The identification of these locations, where gene flow may already be providing genetic variation for future adaptation, illustrates how environmental surrogates for adaptive potential may sometimes fall short in informing conservation planning.

Gene flow among populations can increase the speed of adaptation to warmer conditions, but it can also reduce nonclimatic local adaptations and therefore reduce population fitness and evolvability (37). Given these limitations, it is not surprising that evidence of evolutionary rescue in the wild is rare, although this may be at least partially due to logistical difficulties in obtaining both population dynamics and evolutionary change data (38). Nevertheless, given the rapid rates of climate change relative to rates of evolutionary response, long-lived organisms may struggle to evolve fast enough to keep up with changes (39) unless rates of gene flow from already adapted populations are high enough to allow adaptive variation to rapidly spread through climatically maladapted populations. This stresses the importance of approaches like the one developed in this study that can identify landscape barriers to gene flow between climatically adapted and maladapted populations and therefore can advise on how species should be managed to increase population connectivity.

### Conclusions.

Using a combination of population genomics, spatial ecology, and predictive modeling we show the importance of incorporating genomic data into climate change forecasts. Local adaptations can be a major determinant of the adaptive capacity of populations to changing climatic conditions (3) and therefore should not be ignored in climate change vulnerability assessments (11). Greater intraspecific than interspecific climatic niche dissimilarities highlight the need to account for intraspecific differences in climatic tolerance when forecasting impacts of future climate change. Our study provides an unprecedented example where climate-adaptive genetic variation is directly incorporated into ENMs, rather than only using local adaptations as a measure of sensitivity (14) or relying on neutral population structure as a proxy for intraspecific adaptive variation (12). We show that considering adaptive genetic variation can reduce range loss projections, indicating that current forecasts of extinction risk from climate change are likely an overestimation of the proportion of species committed to extinction. An exception may be species with limited adaptive variation or genetic constraints that have limited capacity to show an adaptive response. Dispersal limitations and increased potential for interspecific competition when considering adaptive genetic variation, due to increased projected future range overlap, stress the role of biotic interactions in limiting species range shift and the persistence of climatically maladapted (or less adapted) species. The fate of populations at the trailing (equatorward or low elevation) edge will depend on the species’ physiological maximum thermal tolerance, while what will happen in areas that will become unsuitable for cold–wet-adapted individuals but suitable for hot–dry genotypes will depend on gene flow from hot–dry-adapted populations. As the example of our forest bats shows, the survival of maladapted populations may be possible through evolutionary rescue, but evolutionary rescue depends not only on individual adaptive capacity but also on landscape connectivity. As such, climate-adaptive conservation management should consider local climatic adaptations and focus not only on areas with threatened populations but also on facilitating movement between populations.

## Materials and Methods

### Generating the Genomic Datasets.

This study was approved by the University of Southampton Ethics Committee. Bats were sampled (nonlethal wing biopsies) between 2010 and 2015 (the majority of samples were taken after 2013) from locations across the species’ ranges in the Iberian Peninsula, southern France, and northern Italy (*SI Appendix*, Tables S6 and S7). The final *M. escalerai* dataset included 220 bats from 67 locations, 18 of which represent colonies (7–10 individuals sampled from cave roosts). The *M. crypticus* dataset included 58 bats from 48 locations (*SI Appendix*, Fig. S5).

Double digest restriction-site-associated DNA sequencing (40) was used to generate a genomic dataset containing tens of thousands of anonymous genetic loci from across the species genomes (41). The final dataset for *M. escalerai* included 18,356 SNPs, 216 individual bats, and a genotyping rate of 0.906. The final dataset for *M. crypticus* included 20,750 SNPs, 57 individual bats, and a genotyping rate of 0.894 (see Datasets S2 and S3 and *SI Appendix* for library preparation and bioinformatics).

### Identifying Climate-Adaptive Genotypes and Individuals.

We carried out a GEA analysis to identify a signature of climate-driven genetic variation based on associations between allele frequencies and local conditions. We focused on two ecologically relevant climatic variables, maximum temperatures of the warmest month and precipitation of the warmest quarter (Bio5 and Bio18, downloaded from WorldClim, www.worldclim.org). GEA analysis was performed with the latent factor mixed model approach (42) and a redundancy analysis (RDA) (43) (see *SI Appendix* for running procedures). We used a conservative approach (21), whereby only SNPs that were identified as being under climate-driven selection for either climatic variable by both GEA methods were classified as climate-adaptive SNPs. RDA was used to plot the spread of individuals in the ordination space based on their climate-adaptive SNPs relative to the maximum temperature and summer rainfall axes (*SI Appendix*).

### Modeling Range Losses Under Future Climate Change.

ENMs were run using the ensemble modeling approach in the R package biomod2 version 3.3-7 (44). Models were replicated 10 times (five for models with low sample sizes, *n* < 50) using the cross-validation approach. Model performance was evaluated based on total ensemble model area under the receiver operator curve scores, TSS, and comparison with null models (see *SI Appendix* for ENM running procedures).

The study extent was set as around 500 km north of the known range limit of *M. crypticus* (the species with the larger range size) to include areas within the theoretical dispersal ability of the species by the end of the century (45). Cell size was set at 30 arc s (∼1 km). Models included bioclimatic variables (downloaded from WorldClim), a static topographic variable that is independent of temperature changes (slope, generated from the Shuttle Radar Topography Mission (SRTM) altitude map, https://www2.jpl.nasa.gov/srtm/), and distance to karsts (Karst Regions of the World) (46) because *M. escalerai* primarily roosts in caves and mines. We removed autocorrelated variables (*R* > 0.75) and variables that did not contribute to model gain (see *SI Appendix*, Table S2 for final model variables). Models were projected to the future (2070) using three ceneral circulation models [Hadley Centre Global Environment Model version 2 Earth Systems model (HadGEM2_ES), Institut Pierre-Simon Laplace Coupled Model 5th Assessment Low Resolution (IPSL-CM5A-LR), and Max Planck Institute for Meteorology Earth System Model Low Resolution (MPI-ESM-LR)] and two RCP scenarios (47), the worst-case scenario, RCP +8.5 W/m^2^, and the more moderate RCP +4.5 W/m^2^ scenario. For each species or group we ran separate models for each GCM, producing an ensemble of 30–60 models for each RCP scenario that were merged together into a single layer.

ENMs included 313 and 168 genetically confirmed records of *M. escalerai* and *M. crypticus*, respectively (the full datasets), obtained from this study and previous studies of the species (19, 20, 26). We also ran separate models for individuals within each species identified as adapted to hot–dry (*M. escalerai, n* = 19; *M. crypticus, n* = 25) and cold–wet (*n* = 41, 18, respectively) conditions based on our genomic dataset to determine whether their climatic niche is different and whether they will be affected differently by future climate change. We calculated extent of overlap in geographic and ecological space (range and niche overlap; *SI Appendix*).

### Landscape Genetics and Evolutionary Rescue Analyses.

The landscape genetics analysis for *M. escalerai* was carried out at the population level (18 populations, *n* = 162), while for *M. crypticus* it was carried out at the individual level, retaining a single sample (the first sample) from each location (*n* = 47). The extent of the analysis was set as the respective species’ ranges. Landscape variables (including habitat suitability, forest cover, land cover, topographic, and climatic variables) were converted to resistance cost surfaces in ArcGIS and assigned costs ranging from 1 (no resistance to movement) to 100 (strong barrier to movement) (*SI Appendix*, Table S8). Circuitscape version 4.0.5 (48) was used to calculate resistance distance matrices between populations or individuals and estimate potential movement pathways across the landscape based on the cumulative cost of movement due to landscape resistance. Although bats are capable of flight, the studied species have relatively limited dispersal ability (49) and therefore are more likely to have a landscape-mediated population structure (50). Potential for evolutionary rescue was determined according to the potential for gene flow from hot–dry-adapted to cold–wet-adapted populations and individuals based on the effect of the landscape on current patterns of genetic differentiation (*SI Appendix*).

### Data Availability.

The raw sequence data from this study have been deposited at the European Nucleotide Archive (ENA), accession no. PRJEB29086 (41). Final SNP datasets for the two species in Genepop format are given in Datasets S2 and S3.

## Supplementary Material

Supplementary File

Supplementary File

Supplementary File

Supplementary File

Supplementary File
