# Supplementary material for: Considering adaptive genetic variation in climate change vulnerability assessment reduces species range loss projections
Source: Proc Natl Acad Sci U S A. 2019 May 6;116(21):10418–23. doi: 10.1073/pnas.1820663116 (PMC6535011; doi:10.1073/pnas.1820663116)
Supplement: Supplementary File [file pnas.1820663116.sapp.pdf]

PNAS
www.pnas.org

Supplementary Information for

Considering adaptive genetic variation in climate change vulnerability assessment reduces species range loss projections

Orly Razgour, Brenna Forester, John Taggart, Michaël Bekaert, Javier Juste, Carlos Ibáñez, Sébastien J. Puechmaille, Roberto Novella-Fernandez, Antton Alberdi, Stephanie Manel

Orly Razgour
Email: Orly.Razgour@soton.ac.uk

This PDF file includes:

Supplementary text: Results
Supplementary text: Materials and Methods
References for SI reference citations
Figs. S1 to S8
Tables S2 to S10

Other supplementary materials for this manuscript include the following:

Supplementary Tables S1 and S11
Datasets S1 to S2

Supplementary text: Results

Genomic datasets

Myotis escalerai: SNPs=18,356; individuals=216; genotyping rate=0.906.
Myotis escalerai neutral: SNPs=17,289; individuals=216; genotyping rate=0.906.
Myotis crypticus: SNPs=20,750; individuals=57; genotyping rate=0.894.
Myotis crypticus neutral: SNPs=20,693; individuals=57; genotyping rate=0.894.

Population structure results

M. escalerai was divided into 3 (PCA) – 4 (snmf) main population clusters, with the main split being between the eastern Pyrenees samples and the rest of the species Iberian range. The remaining 2-3 clusters split Iberian into eastern and western populations, and a separate south-western population (for four clusters) (SI Fig. S7). *M. crypticus* was divided into 4 (PCA) or 3 (snmf) geographical separate population clusters, including the north Iberian Atlantic coast, the Pyrenees, southern France and Italy (SI Fig. S8).

Genotype-Environment Associations analysis results

For *M. escalerai*, LFMM identified 204 loci associated with maximum temperatures and 223 associated with rainfall of the warmest quarter (84 duplicates associated with both variables). Of these loci, 37 and 116, respectively, were also identified as under directional selection by Bayescan. RDA identified 83 loci associated with maximum temperatures and 10 with summer rainfall, 28 and 4 of which overlapped with LFMM, respectively. Combining the two GEA methods results and the two climatic variables, we identified 32 climate-adaptive SNPs that overlapped between the two methods. For *M. crypticus*, LFMM identified 34 loci associated with maximum summer temperatures and 129 loci associated with rainfall of the warmest quarter (16 duplicates). Of these loci, 0 and 1, respectively, were also identified as under directional selection by Bayescan. RDA identified 34 loci associated with maximum temperatures and 119 with summer rainfall, 7 and 31 of which overlapped with LFMM, respectively. Combining the two GEA methods results and the two climatic variables, we identified 38 climate-adaptive SNPs that overlapped between the two methods. The correlation between the environmental predictors was slightly higher than the preferred threshold for *M. escalerai* (r=-0.80), but we retained both predictors due to their ecological relevance. For *M. crypticus* the correlation between the environmental predictors (r=-0.69) was below the preferred threshold.

Landscape genetics analysis results

Genetic differentiation between the 18 *M. escalerai* colonies (N=162) was relatively high but varied considerably (neutral dataset: mean Fst=0.117 ±0.08, range: 0.005-0.328; full dataset: mean Fst=0.131 ±0.08, range: 0.005-0.359; SI Table S10). Highest levels of differentiation were found between the two eastern Pyrenees populations (France and Girona) and all other populations, while lowest levels were between Caceres and all populations apart from the Pyrenees and north-eastern populations, and between the Portuguese populations. Similarly, genetic differentiation among *M. crypticus* individual locations (N=47) was variable (neutral dataset: mean Euclidian genetic distance=111.7 ±26.9, range: 56.2-153.1), with highest rates found between some of the French and Italian locations and Iberian locations (SI Table S11).

In *M. escaleari* neutral genetic distance was most strongly related to slope (R^2=0.76). After accounting for the effect of geographic distance, the strongest correlated variable on its own was slope (R^2=0.383), but the strongest relationship was with the combination of slope and tree cover (R^2=0.532) (Table S4). Similarly, in *M. crypticus* – genetic distance was most strongly related to forest cover (variable lcfor100; R^2=0.791). After accounting for the effect of geographic distance, the strongest correlated variable on its own was forest cover (R^2=0.316), but the strongest relationship was with the combination of forest cover and slope (R^2=0.356) (Table S5).

Supplementary text: Materials and Methods

Generating the genomic datasets

Genomic DNA was extracted from all samples using the Qiagen DNeasy Blood and Tissue extraction kit, and was quantified with Qubit® Fluorometer 2.0 and the dsDNA High Sensitivity assay kit (Invitrogen, ThermoFisher Scientific). DNA quality was assessed through visualisation on 1% agarose gels. Samples with low DNA quantity (<2 ng/µL) or quality were excluded. ddRAD library preparation protocol was based on the methodology in Peterson et al.(1), with modifications from Manousaki et al.(2). DNA samples were simultaneously digested by two high fidelity restriction enzymes: SbfI (CCTGCA|GG recognition site), and NlaIII (CATG| recognition site) (New England Biolabs, UK).

Bioinformatics of the high-throughput sequencing data was carried out in STACKS v1.46(3). We first did a reference-based alignment using the reference genome of two congeners, *Myotis davidii* and *Myotis branditii*, to obtain knowledge of the allelic diversity before setting up the parameters for the *de novo* approach used to call SNPs. We ran STACKS for *de novo* assembly with the following optimised parameters: a minimum stack depth [m] of 6, a maximum of 2 mismatches allowed in a locus [M]) in an individual and maximum of 1 mismatches between loci when building the catalog [n]). For the 312 samples, belonging to the two species, we got 724,579 unique RAD-tags and 86,051 shared RAD-markers (Allele=2, SNP=1-3, Coverage=10+ samples per marker, Read number >6 per samples). We exported one SNP (randomly selected) per RAD-marker. The mean coverage of each SNP selected was 63 (range 19-248). The SNP data set was processed in PLINK v1.9(4). To improve data robustness, only RAD-markers that were genotyped in at least 75% of the samples and had minor allele frequencies above 0.03 were considered for analysis. We also removed individuals that had more than 50% missing data and close relatives (based on identity-by-state distances, PI HAT >0.5).

Identifying climate-adaptive genotypes and individuals

Outlier tests were performed in Bayescan(5) to identify SNPs potentially under directional selection, or linked with genes under selection (1,000,000 iterations, 50,000 burn-in and 20 pilot runs; false discovery rate was set to 0.05). Outlier tests were performed on the M. escalerai population dataset (18 sampled populations, N=162) and the whole *M. crypticus* dataset divided into four distinct geographic and genetic population clusters based on Principle Component Analysis, performed in the R package Adegenet 2.0.0(6). SNPs identified as under directional selection (1237 in *M. escalerai* and 271 in *M. crypticus*) were removed from the analyses of population structure and neutral patterns of genetic differentiation.

Missing genotypes were imputed using a sparse non-negative matrix factorisation algorithm(7) as implemented in the R package LEA v2.0.0 using function snmf(8). Snmf imputes genotypes from the estimated ancestry coefficients and ancestral genotype frequencies. Genetic population structure was determined at the individual level based on a combination of ordination methods (Principle Component Analysis, PCA, performed in the R package Adegenet 2.0.0(6) and ancestry coefficient estimations performed using the snmf function(8), a computationally fast algorithm similar to STRUCTURE. Number of population clusters was set at K=1-10, using 10 replicates for each K and the entropy function.

Genotype-Environment Associations analysis. We focused on two climatic variables that are likely to directly affect bat survival and reproductive success. Increased aridity and prolonged droughts around the Mediterranean are predicted to affect insect prey availability during the summer(9) and thus decrease reproductive success in bats(10) that breed during the summer and rely on insect prey. Increased maximum summer temperatures have already been implicated in mass mortality events of bats(11). In order for bats to survive in warmer and more arid conditions they requires physiological adaptations to reduce evaporative water loss(12).

For *M. escalerai* we ran LFMM(13) with K=3-4, and for *M. crypticus* with K=2-4. Final K values were determined based on the genomic inflation factor (lambda values closest to 1): K=4 for *M. escalerai* and K=3 for *M. crypticus*. We performed five LFMM repetition runs with 10,000 iterations and 5000 burn-in. Z-scores of multiple runs were combined using the median value and p-values were adjusted for expected FDR of 0.05 (following the procedures in Frichot & François(8)). For both species, we ran an RDA on the scaled genotypes and environmental predictors. We assessed the significance of the full RDA and each constrained axis using 999 permutations(14). We then identified outlier loci as those markers with a locus score ±3 standard deviations from the mean score of each of the two constrained axes(15).

Modelling range losses under future climate change

Ecological niche models running procedures. When running biomod2(16), we initially included six commonly used ENM techniques (Maximum Entropy (Maxent v.3.4.1(17)), Classification Tree Analysis (CTA), Generalised Boosting Model (GBM), Flexible Discriminant Analysis (FDA), Random Forest (RF) and Artificial Neural Network (ANN)), but only retained the first two techniques due to poor performance of the remaining models with the smaller sample size datasets. Pseudo-absences were selected from the model extent. We used ENMTools v1.3(18) to select the number of model features and regularisation values for the Maxent models based on AIC scores (AICc for models with N<100; Table S1 for final model features). Model AUC scores were compared to 100 null models, generated in ENMTools through resampling the altitude layer, to determine whether models performed significantly better than random(19).

Range and niche overlap. Extent of overlap in geographic space (range overlap) was measured in ArcGIS 10.3.1 (ESRI) based on the binary model outputs (binary suitable/unsuitable maps were generated based on the threshold value that minimises the difference between sensitivity and specificity). Overlap in ecological space was calculated using Schoener's D index of niche overlap in ENMTools. Identity tests carried out on 30 simulated datasets were used to determine whether overlap was significantly higher or lower than expected by chance.

Landscape genetics analysis methods

Genetic differentiation between *M. escalerai* populations (Fst) was computed with the R package diveRsity v1.9.90(20). Genetic differentiation between individual bats was determined using the Euclidean genetic distance measure in the R package Adegenet v2.0.0(6). Both analyses were carried out on neutral datasets after excluding outlier (Bayescan) and climate-adaptive (LFMM+RDA) loci.

Generating landscape resistance layers. The following landscape variables were included in the analysis: habitat suitability based on ENMs; percent tree cover (percent tree canopy cover map(21)); distance to broadleaf forest, conifer forest and all forests (generated from GlobCover2009 map [European space Agency, http://due.esrin.esa.int/page_globcover.php] using the Euclidian distance tool in ArcGIS); land cover-forest (reclassified land cover map into forest=1, and the rest 50-100 [lcfor50/75/100]); land cover type (reclassified GlobCover2009 map based on different costs to all habitats [lc1/lc50/100]); altitude, slope and ruggedness (calculated from SRTM map [https://www2.jpl.nasa.gov/srtm/] in ArcGIS); autumn temperatures and autumn rainfall (calculated from WorldClim mean temperatures and precipitation maps for September-November).

Statistical analysis. We carried out a two-step analysis, first correlating each variable against genetic distance to select variables to include in the analysis based on the strength of their correlations with genetic distance. We removed variables that were strongly correlated with Euclidean distance or other variables ($R^2 > 0.56$ [R>0.75]). Second, to partial-out the effect of geographic distance, we correlated the residuals of the correlation between genetic and geographic distance against the selected landscape resistance variables. The best-fit model was selected based on highest R^2 values and significant P values for all variables (P<0.05).

SI References

1. Peterson BK, Weber JN, Kay EH, Fisher HS, Hoekstra HE (2012) Double digest RADseq: An inexpensive method for de novo SNP discovery and genotyping in model and non-model species. *PLoS One* 7(5):e37135.
2. Manousaki T, et al. (2016) Exploring a Nonmodel Teleost Genome Through RAD Sequencing—Linkage Mapping in Common Pandora, Pagellus erythrinus and Comparative Genomic Analysis. *G3 Genes|Genomes|Genetics* 6(3):509–519.
3. Catchen J, Hohenlohe PA, Bassham S, Amores A, Cresko WA (2013) Stacks: An analysis tool set for population genomics. *Mol Ecol* 22(11):3124–3140.
4. Purcell S, et al. (2007) PLINK: A Tool Set for Whole-Genome Association and Population-Based Linkage Analyses. *Am J Hum Genet* 81(3):559–575.
5. Foll M, Gaggiotti O (2008) A genome-scan method to identify selected loci appropriate for both dominant and codominant markers: A Bayesian perspective. *Genetics* 180(2):977–993.
6. Jombart T (2008) Adegenet: A R package for the multivariate analysis of genetic markers. *Bioinformatics* 24(11):1403–1405.
7. Frichot E, Mathieu F, Trouillon T, Bouchard G, François O (2014) Fast and efficient estimation of individual ancestry coefficients. *Genetics* 196(4):973–983.
8. Frichot E, François O (2015) LEA: An R package for landscape and ecological association studies. *Methods Ecol Evol* 6(8):925–929.
9. Frampton GK, Van Den Brink PJ, Gould PJL (2000) Effects of spring drought and

irrigation on farmland arthropods in southern Britain. *J Appl Ecol* 37(5):865–883.
10. Adams RA (2010) Bat reproduction declines when conditions mimic climate change projections for western North America. *Ecology* 91(8):2437–2445.
11. Welbergen JA, Klose SM, Markus N, Eby P (2008) Climate change and the effects of temperature extremes on Australian flying-foxes. *Proc R Soc B Biol Sci* 275(1633):419–425.
12. Muñoz-Garcia A, et al. (2016) Metabolic rate, evaporative water loss and thermoregulatory state in four species of bats in the Negev desert. *Comp Biochem Physiol Part A Mol Integr Physiol* 191:156–165.
13. Frichot E, Schoville SD, Bouchard G, François O (2013) Testing for associations between loci and environmental gradients using latent factor mixed models. *Mol Biol Evol* 30(7):1687–1699.
14. Legendre P, Oksanen J, ter Braak CJF (2011) Testing the significance of canonical axes in redundancy analysis. *Methods Ecol Evol* 2(3):269–277.
15. Forester BR, Lasky JR, Wagner HH, Urban DL (2018) Comparing methods for detecting multilocus adaptation with multivariate genotype–environment associations. *Mol Ecol* 27(9):2215–2233.
16. Thuiller W, Lafourcade B, Engler R, Araújo MB (2009) BIOMOD - A platform for ensemble forecasting of species distributions. *Ecography* 32(3):369–373.
17. Phillips SJ, Anderson RP, Schapire RE (2006) Maximum entropy modeling of species geographic distributions. *Ecol Modell* 190(3–4):231–259.
18. Warren DL, Glor RE, Turelli M (2010) ENMTools: A toolbox for comparative studies of environmental niche models. *Ecography* 33(3):607–611.
19. Raes N, ter Steege H (2007) A null-model for significance testing of presence-only species distribution models. *Ecography* 30(5):727–736.
20. Keenan K, Mcginnity P, Cross TF, Crozier WW, Prodöhl PA (2013) DiveRsity: An R package for the estimation and exploration of population genetics parameters and their associated errors. *Methods Ecol Evol* 4(8):782–788.
21. Hansen MC, et al. (2013) High-resolution global maps of 21st century forest cover change. *Science* 342(6160):850–853.

Supplementary Figures

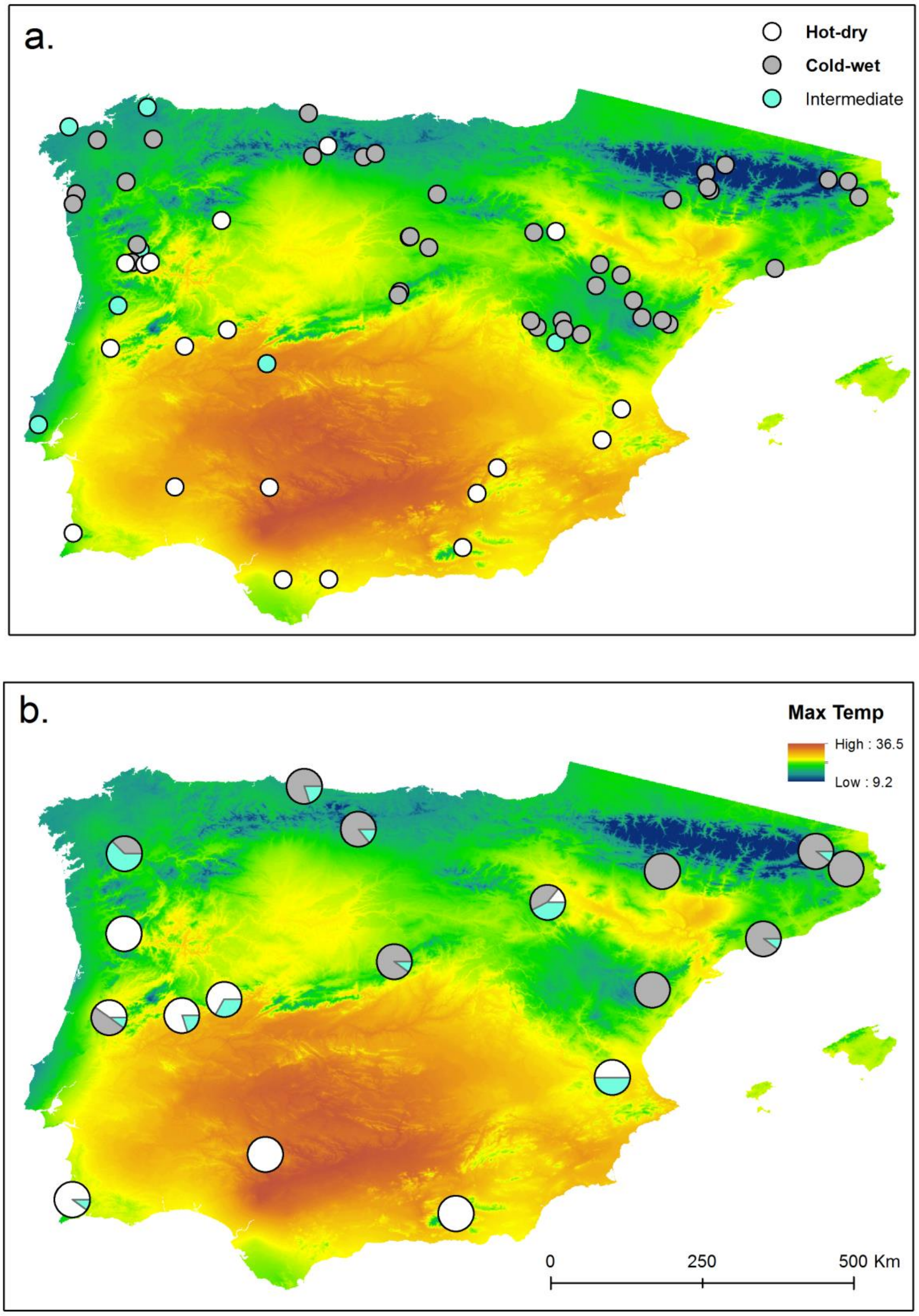

Fig. S1. *M. escalerai* spatial distribution of individuals adapted to hot-dry (white) and cold-wet (grey) conditions and intermediate genotype (light blue) in the individual (a) and population (b) datasets, presented over the maximum temperature map.

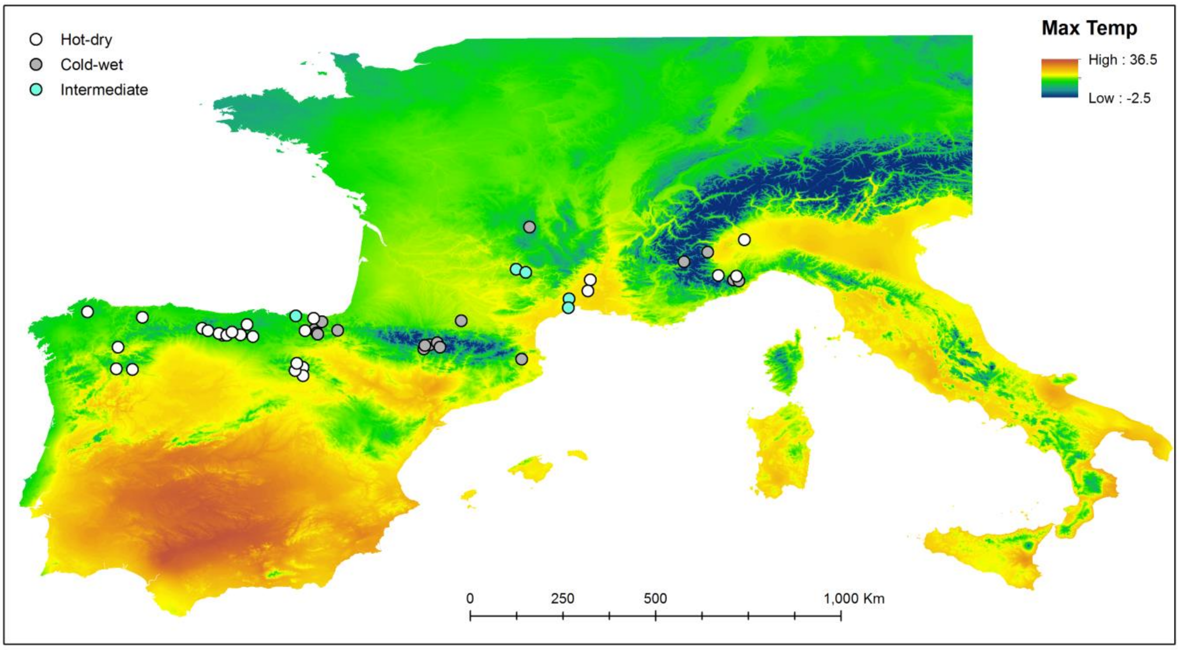

Fig. S2. *M. crypticus* spatial distribution of individuals adapted to hot-dry (white) and cold-wet (grey) conditions and intermediate genotype (light blue), presented over the maximum temperature map.

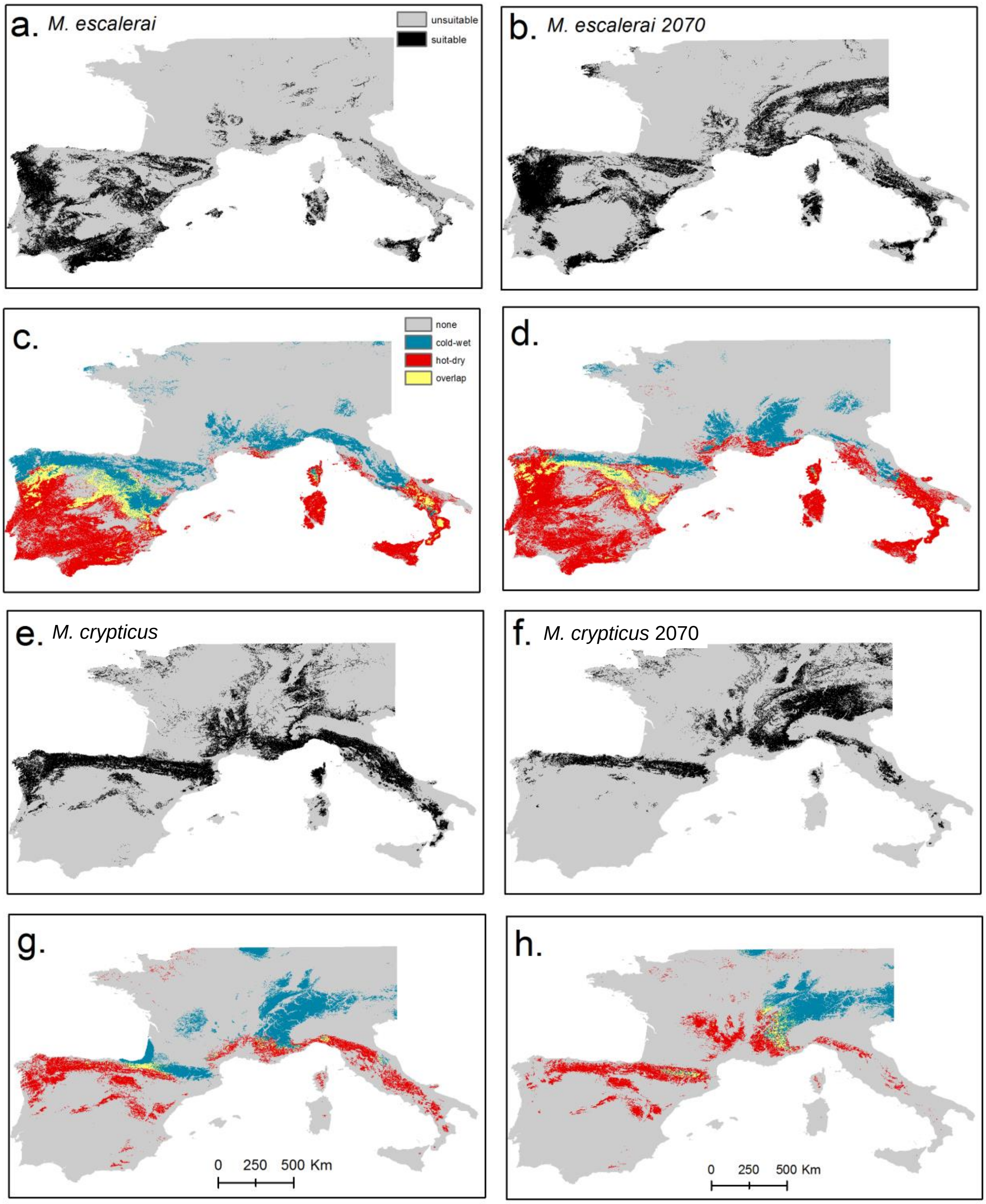

Fig. S3. Ecological niche modelling outputs for *M. escalerai* (a-d) and *M. crypticus* (e-h), including the full dataset (a-b, e-f) and overlap between separate models for individuals adapted to hot-dry (red) and cold-wet (blue) conditions (c-d, g-h), under present (a,c,e,g) and future (2070, RCP 4.5) climatic conditions (b,d,f,h).

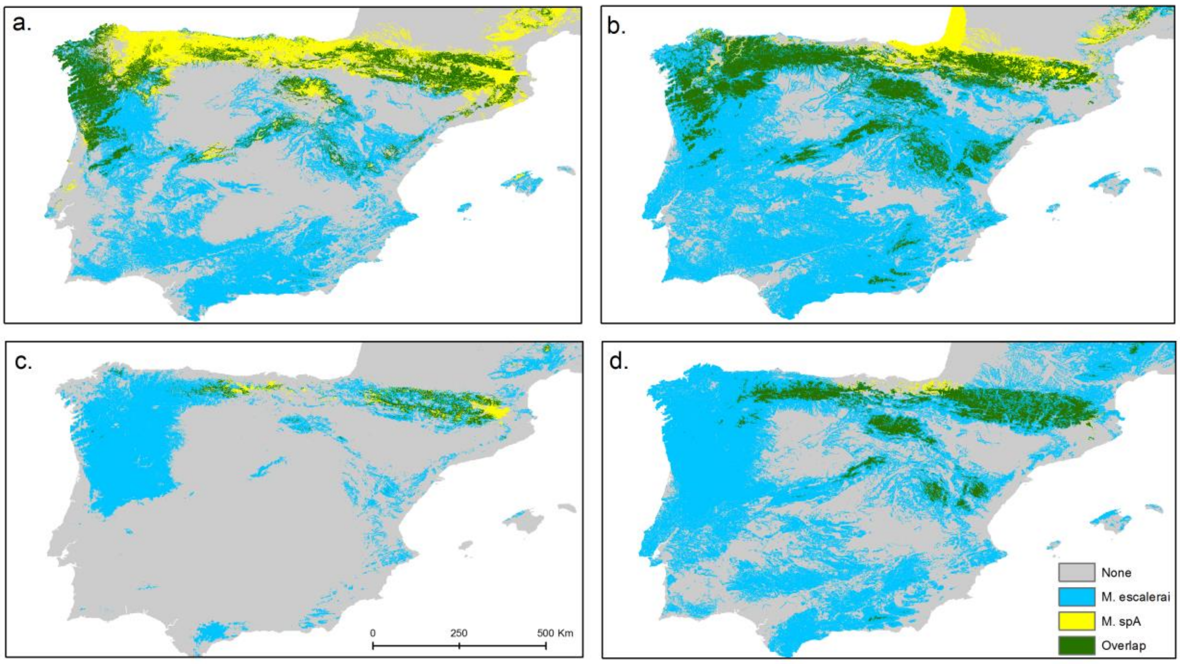

Fig. S4. Overlap between Species distribution modelling projection of *M. escalerai* and *M. crypticus* (*M. spA*), including the full dataset (a, c) and the combined output of models ran separately for individuals adapted to hot-dry and cold-wet conditions (b,d), under present (a-b) and future (2070, RCP 8.5) climatic conditions (c-d).

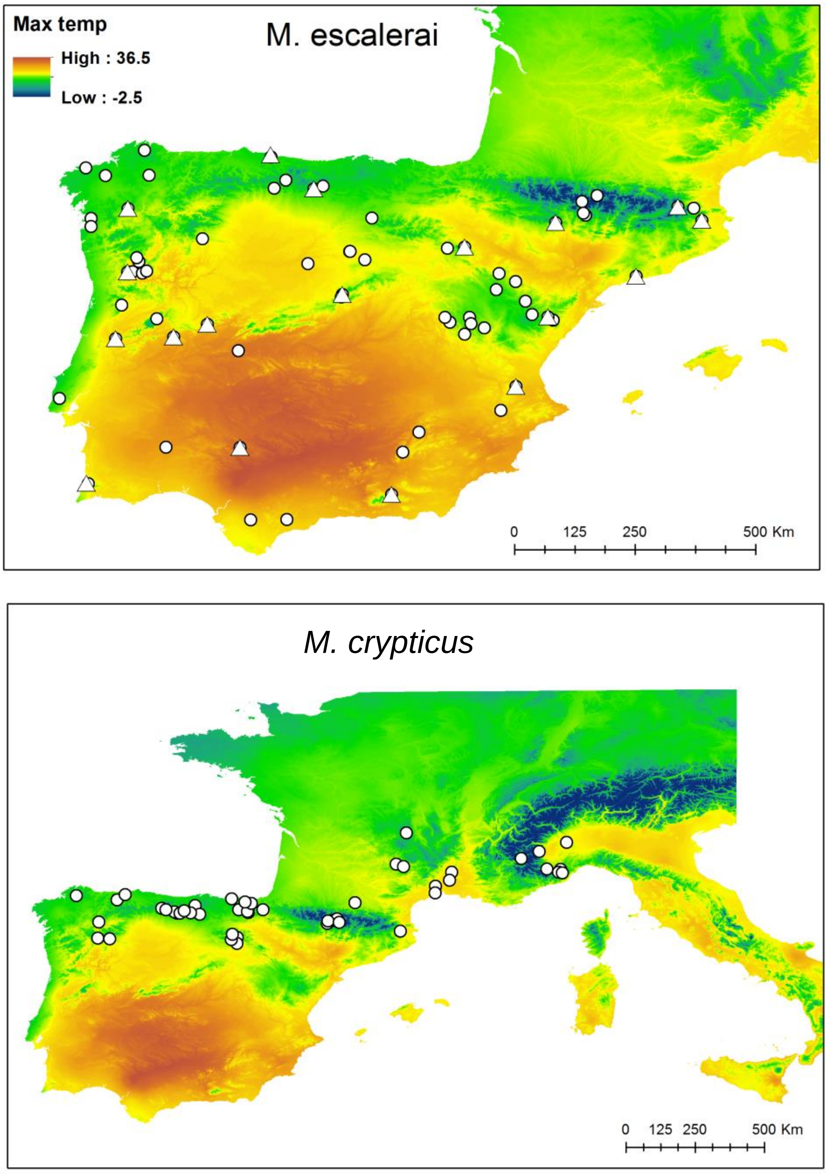

Fig. S5. Sampling locations for *M. escalerai* and *M. crypticus* displayed over a map of maximum temperatures, ranging from low in blue to high in red. Circles denote locations with 1-2 samples, while triangles denote colony sites with 7-10 samples.

a.

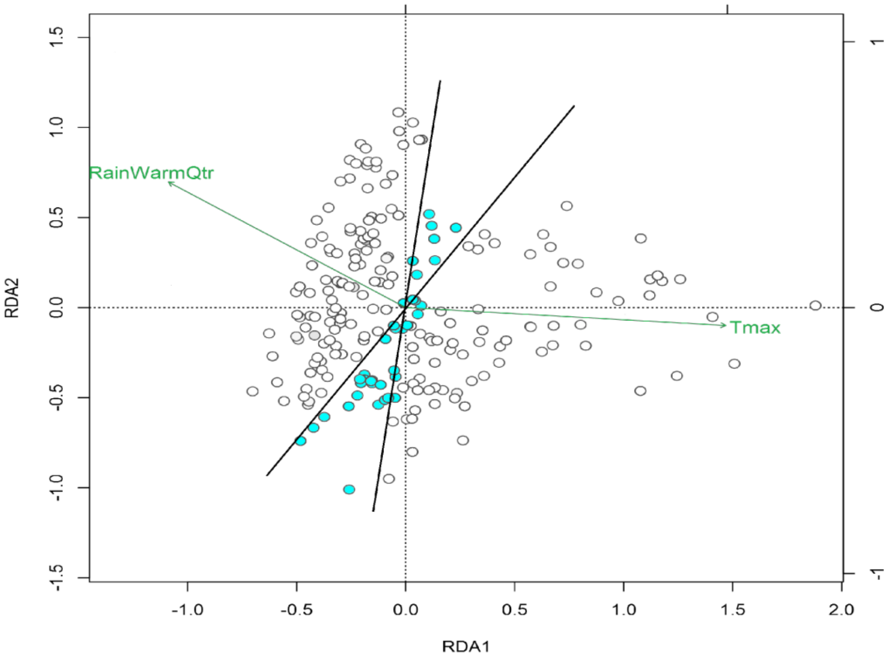

b.

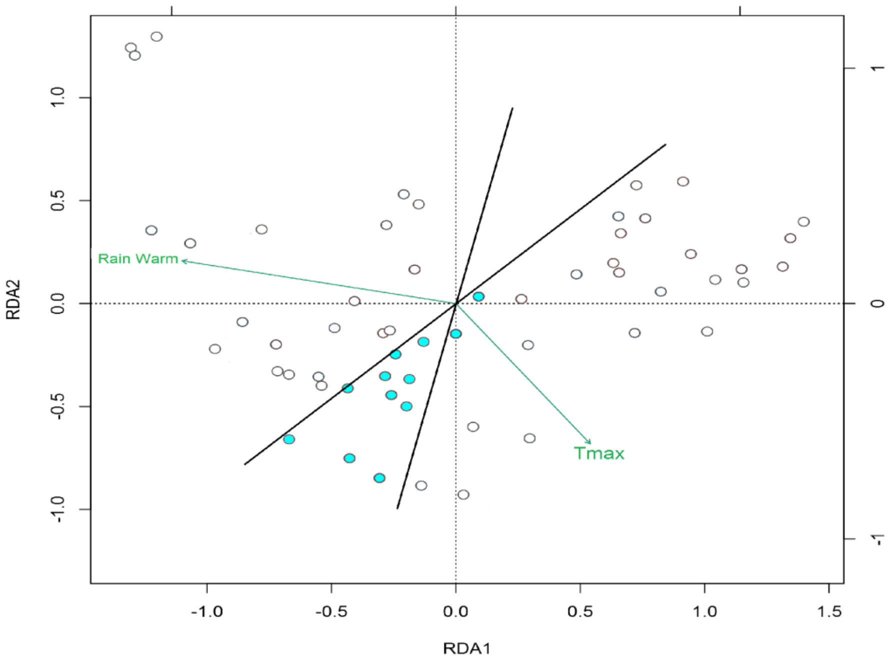

Fig. S6. RDA plots of the genotypes of individual *M. escalerai* (a) and *M. crypticus* (b) bats falling across the ordination space relative to the maximum temperature and rainfall of warmest quarter axes. Individuals falling within the high temperature and low rainfall space were classified as hot-dry adapted, while those falling within the low temperature and high rainfall space were classified as cold-wet adapted. Individual genotypes falling in the centre, not along either of the environmental vectors (marked in blue) were classified as intermediate. Black bold lines are the perpendicular lines relative to where each environmental axis crosses the centre (zero point) of the ordination space.

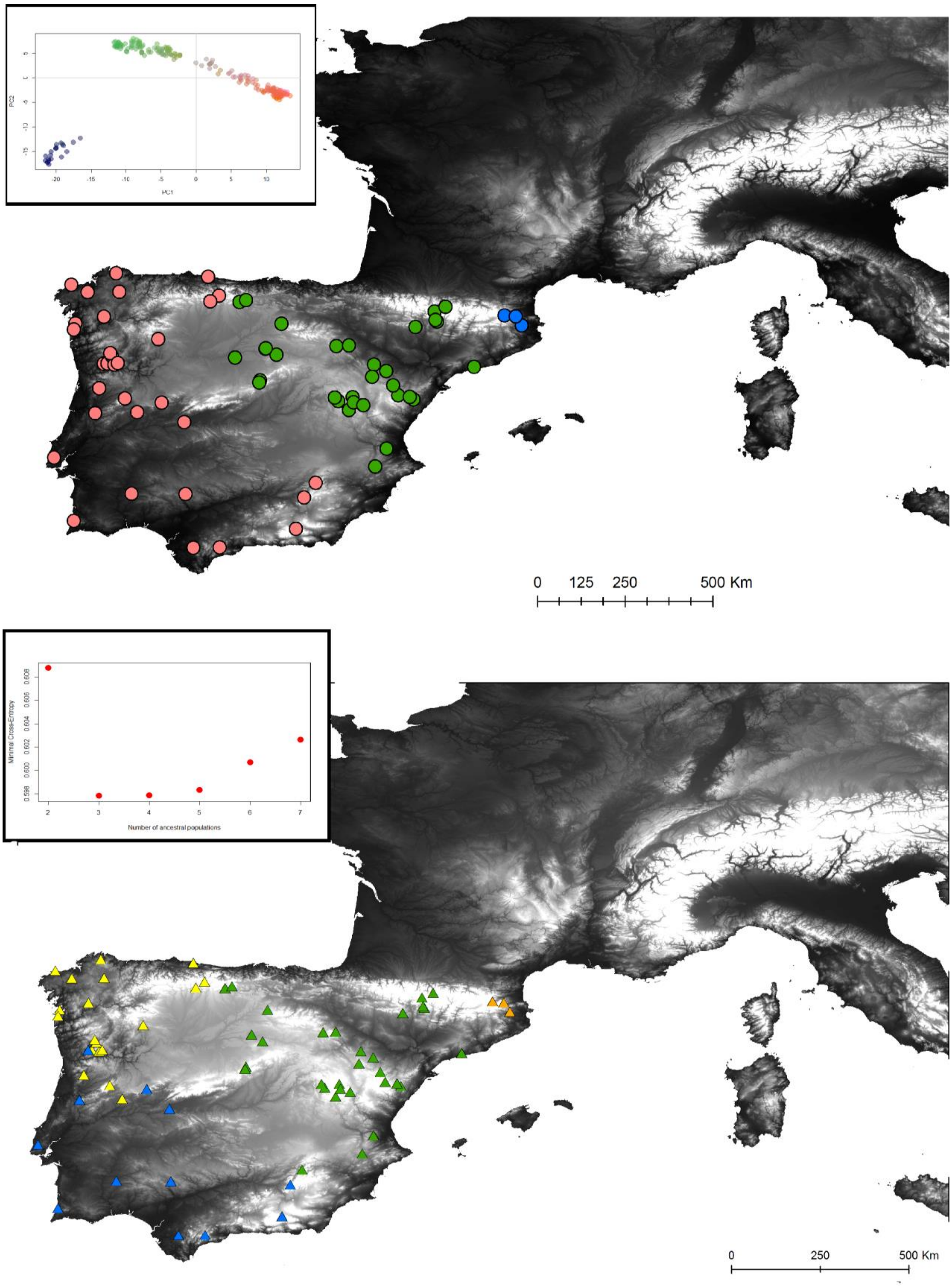

Fig. S7. *Myotis escalerai* population structure presented over an altitude map, based on a) PCA, dividing the neutral dataset into three geographically separate population clusters, and b) snmf assignment tests, with minimal cross entropy plot indicates 3-4 population clusters.

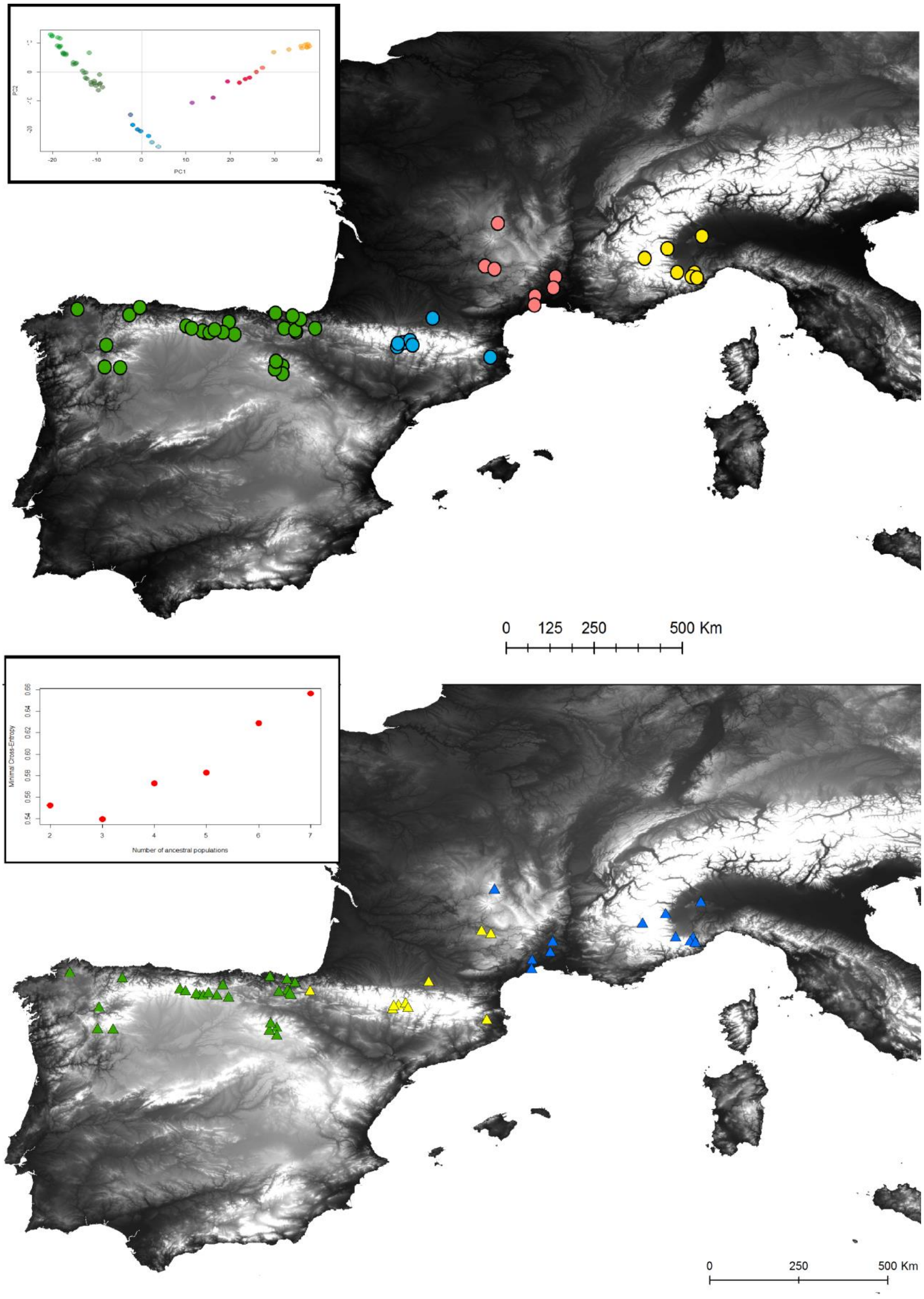

Fig. S8. *Myotis crypticus* population structure presented over an altitude map, based on a) PCA, dividing the neutral dataset into three-four geographically separate population clusters, and b) snmf assignment tests, with minimal cross entropy plot indicates three population clusters.

Supplementary Tables

Table S2 – Results of the analysis starting with 75% and 50% subsets of the SNP datasets compared to the full SNP dataset, with range of results based on different GCMs in brackets (Mesc= *M. escalerai*; Mcry=*M. crypticus*).

	**full dataset ~20,000 SNPs**	**75% dataset ~15,000 SNPs**	**50% dataset ~10,000 SNPs**
**% range loss hot+cold Mesc**	18.5 (12.6-25.1)	17.4 (8.9-26.1)	8.05 (0.7-20.4)
**% range loss hot+cold Mcry**	58.52 (44.3-68.1)	71.69 (59.6-74.3)	65.51 (54.3-72.6)
**% range loss cold Mesc**	58.73 (53.1-77.6)	43.33 (36.4-67.6)	52.51 (44.8-73.9)
**% range loss cold Mcry**	99.96 (77.9-100)	98.31 (77.5-100)	99.91 (82.9-100)
**% range overlap changes**	5.45 (3.9-7.9)	5.45 (4.8-8.3)	6.75 (4.8-6.8)

Table S3. Results of the ensemble Ecological Niche Models, including number of records used in the models (N), final Maxent model parameters (regularization [R] and features [f]), variable contribution to the models with most important variables highlighted in bold, percent of the study area predicted to be climatically suitable under present (% suitable) and future (2070, rcp8.5) conditions and percent range changes (rcp8.5 across the whole range and rcp4.5 for Iberia only). (BIO5=maximum temperature of the warmest month, BIO6=minimum temperature of the coldest month, BIO17=rainfall in the dry quarter, BIO12=annual rainfall, BIO15=rain seasonality, BIO16=rainfall in the wet quarter, BIO7=temperature annual range, BIO18=rainfall in the warmest quarter, April Tmin=minimum April temperature).

Taxa	N	Maxent Model	BIO5	BIO6	BIO7	BIO12	BIO15	BIO16	BIO17	BIO18	April Tmin	slope	karsts	% present	% future rcp8.5	% range change rcp8.5	% range change Iberia rcp4.5
M. escalerai all	313	R1, f4	2.8	7.8	7.5	2.7	**43.4**					**32.1**	3.8	16.99	15.03	11.68	16.25
M. escalerai hot-dry	19	R1.5, f4			0.1	1.6	**42.3**			**39.8**	3.8	12.5		21.93	29.56	-34.52	-6.21
M. escalerai cold-wet	41	R1.5, f4					6.4	10.3		**41.5**	16.5	24.1	1.3	16.15	10.84	32.90	48.37
M. crypticus	168	R1.5, f4	0.2	9.2	10.8	15.3	10.4					**54.1**		19.89	8.46	57.57	55.17
M. crypticus hot-dry	25	R1, f2			4.1	12.9	5.1			**31.7**	**28.5**	17.8		8.75	6.66	24.03	17.25
M. crypticus cold-wet	18	R2, f4		5.1	9.8			0.1	**49.4**			**35.6**		8.97	5.27	41.31	82.74

Table S4. Niche overlap Results (Schoener's D) top triangle, and niche identity test results 99% CI in bottom triangle. Niche overlap values that are significantly lower than random based on the niche identity test are marked with *.

	M. escalerai	***M. escalerai* cold**	***M. escalerai* hot**	***M. crypticus***	***M. crypticus* cold**	***M. crypticus* hot**
M. escalerai		0.576*	0.724*	0.480*	0.325*	0.440*
***M. escalerai* cold**	0.810-0.836		0.432*	0.584*	0.452*	0.689*
***M. escalerai* hot**	0.768-0.800	0.729-0.790		0.277*	0.138*	0.369*
M. crypticus	0.845-0.869	0.742-0.781	0.680-0.712		0.624*	0.578*
***M. crypticus* cold**	0.757-0.787	0.558-0.612	0.738-0.800	0.674-0.731		0.465*
***M. crypticus* hot**	0.783-0.809	0.739-0.799	0.722-0.773	0.721-0.757	0.697-0.764	

Table S5. Multiple Regressions on Distance Matrices between genetic differentiation (Fst) and landscape resistance variables in the *M. escalerai* population dataset, after the removal of variables correlated with Euclidean distance. The most important variables are highlighted in bold and auto-correlated variables are colour-coded. (euc-distance=Euclidean distance [isolation by distance]; lcfor=forest variables derived from land cover map; landcov=land cover; rain=rainfall during September-November; ENM=ecological niche model including land cover variables; NS: P>0.05)

Variable	Partialed-out variable	R^2	F	P
euc_dist		0.495	147.9	0.0001
altitude		0.43	113.8	0.0001
autumn		0.642	270.4	0.0001
conifer		0.04	5.8	0.017
lcfor100/75/50				NS
landcov1		0.147	26	0.0001
landcov2		0.114	19.4	0.0001
landcov3		0.05	7.8	0.006
rain3		0.231	45.5	0.0001
rain6		0.144	25.4	0.0001
rugged		0.551	185.4	0.0001
ENM		0.379	92	0.0001
**slope**		**0.76**	**477.4**	**0.0001**
tree cover		0.17	30.9	0.0001
ENM	euc_dist	0.08	13.1	0.001
altitude	euc_dist	0.116	19.7	0.0001
rain3	euc_dist	0.086	14.3	0.0007
**slope**	**euc_dist**	**0.383**	**93.8**	**0.0001**
tree	euc_dist	0.024	3.6	0.058
ENM+slope	euc_dist	0.405	51.1	0.0001
ENM+rain	euc_dist			NS
ENM+altitude	euc_dist	0.152	13.4	0.0001
ENM+tree	euc_dist	0.342	38.9	0.0001
**slope+tree**	**euc_dist**	**0.532**	**85.3**	**0.0001**
slope+altitude	euc_dist			NS
slope+rain	euc_dist			NS
slope+tree+altitude	euc_dist			NS
ENM+slope+tree	euc_dist	0.56	63.2	0.0001

Table S6. Multiple Regressions on Distance Matrices between genetic differentiation (Fst) and landscape resistance variables in the *M. crypticus* dataset, after the removal of variables correlated with Euclidean distance. The most important variables are highlighted in bold and auto-correlated variables are colour-coded. (euc-distance=Euclidean distance [isolation by distance]; lcfor=forest variables derived from land cover map; landcov=land cover; rain=rainfall during September-November; NS: P>0.05).

Variable	Partialed-out variable	R2	F	P
euc_dist		0.668	2167	0.0001
altitude		0.26	379.9	0.0001
lcfor75		0.76	3421.4	0.0001
**lcfor100**		**0.792**	**4112.2**	**0.0001**
landcov3		0.634	1869	0.0001
rain6		0.38	662.5	0.0001
slope		0.361	608.4	0.0001
**lcfor100**	**euc_dist**	**0.316**	**499.4**	**0.0001**
slope	euc_dist			NS
rain6	euc_dist	0.01	10.9	0.022
**lcfor100+slope**	**euc_dist**	**0.356**	**298.5**	**0.0001**
lcfor100+rain	euc_dist	0.349	288.5	0.0001
slope+rain	euc_dist			NS
lcfor100+slope+rain	euc_dist			NS

Table S7. *Myotis escalerai* samples included in the final genomic dataset and number of samples included from each location. The first 18 locations represent populations with >6 samples (coordinates in WGS1984). Coordinates were rounded up to one decimal point to protect sensitive sites.

Location	Country	N	Year	LAT	LONG
Asturia	Spain	10	2015	43.5	-5.3
Girona	Spain	10	2015	42.3	2.7
France-CAN	France	9	2013	42.5	2.3
Litoral	Portugal	10	2015	40.1	-8.2
Algarve	Spain	10	2015	37.4	-8.8
Minho	Portugal	10	2015	41.3	-8.0
Baixa	Portugal	10	2015	40.1	-7.2
Zaragoza	Spain	7	2015	41.8	-1.7
Galicia	Spain	8	2015	42.5	-8.0
Catalunya	Spain	10	2015	41.2	1.5
Granada	Spain	9	2015	37.2	-3.1
Sevilla	Spain	10	2015	38.0	-5.9
Segovia	Spain	10	2015	40.9	-4.0
Palencia	Spain	7	2014	42.9	-4.5
Caceres	Spain	9	2010	40.3	-6.5
Huesca	Spain	7	2013	42.2	0.0
Castellón	Spain	6	2015	40.5	-0.2
Valencia	Spain	10	2015	39.2	-0.8
Teruel A35	Spain	1	2013	40.5	-0.5
Teruel A37	Spain	1	2013	40.4	-0.1
FR-BAT	France	2	2015	42.5	2.5
Beira Alta	Portugal	1	2015	40.4	-7.5
Trás-os-Montes	Portugal	1	2015	41.9	-6.6
Oeste	Portugal	1	2015	39.0	-9.3
Alentejo	Portugal	1	2015	38.0	-7.3
Soria R01	Spain	2	2015	41.8	-2.0
Teruel R18	Spain	1	2015	41.1	-0.8
Zaragoza R19	Spain	1	2015	41.3	-1.1
Teruel R21	Spain	1	2015	41.0	-1.1
Teruel R22	Spain	1	2015	40.8	-0.6
Albacete R24	Spain	1	2015	38.7	-1.1
Cuenca R26	Spain	1	2015	40.1	-1.7
Cuenca R27	Spain	1	2015	40.4	-2.0
Cuenca R28	Spain	1	2015	40.5	-2.1
Teruel R30	Spain	1	2015	40.5	-1.6
Teruel R31	Spain	1	2015	40.3	-1.6
Teruel R32	Spain	1	2015	40.3	-1.4
León SA15	Spain	1	2014	43.0	-5.1
Palencia SA24	Spain	1	2014	42.9	-4.4
Segovia SA28	Spain	1	2013	40.8	-4.0
Cadiz SB26	Spain	2	2011	36.7	-5.7

Caceres SB29	Spain	1	2011	39.8	-5.9
Valladolid SB30	Spain	1	2013	41.5	-4.6
Segovia SB31	Spain	1	2012	40.8	-4.0
Pontevedra SB32	Spain	1	2012	42.3	-8.7
Lugo SB40	Spain	1	2012	43.6	-7.7
Huesca SB42	Spain	1	2012	42.4	0.5
Huesca SB43	Spain	1	2012	42.6	0.5
Huesca SB44	Spain	1	2012	42.7	0.7
Lugo SB46	Spain	1	2013	43.1	-7.6
La Coruna SB47	Spain	1	2013	43.3	-8.8
Huesca SB48	Spain	1	2013	42.4	0.5
Segovia SB60	Spain	1	2014	41.5	-3.6
Burgos SB61	Spain	1	2014	42.3	-3.5
Burgos SB62	Spain	1	2014	41.7	-3.9
Burgos SB63	Spain	1	2015	41.7	-3.9
Lugo SB64	Spain	1	2015	43.1	-7.6
Pontevedra SB66	Spain	1	2015	42.2	-8.8
Leon SB70	Spain	1	2015	42.9	-5.3
Malaga SB72	Spain	1	2015	36.7	-5.0
Cadiz SB74	Spain	1	2015	36.7	-5.7
Jaen SB75	Spain	1	2015	37.9	-2.9
Jaen SB76	Spain	1	2015	38.3	-2.6
Portugal SB77	Portugal	1	2014	40.7	-8.1
Portugal SB78	Portugal	1	2014	41.3	-7.9
Portugal SB82	Portugal	1	2014	41.5	-7.8
Portugal SB84	Portugal	1	2014	41.3	-7.7
Portugal SB86	Portugal	1	2014	41.6	-7.8
Portugal SB87	Portugal	1	2014	41.3	-7.7
Galicia SB92	Spain	1	2014	43.1	-8.4

Table S8. *Myotis crypticus* samples included in the final genomic dataset and number of samples included from each location (coordinates in WGS1984). Coordinates were rounded up to one decimal point to protect sensitive sites.

Sample	Country	N	Year	LAT	LONG
Mcrypticus_M180	France	1	2013	43.2	1.2
Mcrypticus_M244	France	1	2014	44.6	6.6
Mcrypticus_M246	France	1	2014	44.5	2.6
Mcrypticus_M251	France	1	2014	44.4	2.8
Mcrypticus_M301	France	2	2014	45.5	2.9
Mcrypticus_M568	France	1	2014	44.2	4.4
Mcrypticus_M573	France	1	2014	43.7	3.8
Mcrypticus_M575	France	1	2015	43.9	4.3
Mcrypticus_M577	France	1	2015	43.5	3.8
Mcrypticus_M196	Italy	1	2013	44.3	7.5
Mcrypticus_M202	Italy	1	2013	44.3	7.9
Mcrypticus_M277	Italy	2	2014	45.2	8.1
Mcrypticus_M281	Italy	1	2014	44.2	7.8
Mcrypticus_M56	Italy	1	2013	44.9	7.2
Mcrypticus_M94	Italy	2	2012	44.2	8.0
Mcrypticus_A01	Spain	2	2014	43.0	-2.3
Mcrypticus_A03	Spain	1	2014	43.0	-2.6
Mcrypticus_A05	Spain	1	2014	42.9	-2.3
Mcrypticus_A06	Spain	1	2014	42.9	-2.3
Mcrypticus_A07	Spain	3	2014	43.2	-2.2
Mcrypticus_A19	Spain	4	2014	43.3	-2.4
Mcrypticus_A27	Spain	1	2015	43.0	-1.8
Mcrypticus_A31	Spain	1	2015	42.1	-2.6
Mcrypticus_A33	Spain	1	2013	43.3	-2.8
Mcrypticus_C15	Spain	1	2015	42.3	2.7
Mcrypticus_SA01	Spain	1	2015	43.1	-4.0
Mcrypticus_SA02	Spain	1	2015	42.8	-3.8
Mcrypticus_SA03	Spain	1	2015	42.9	-4.1
Mcrypticus_SA06	Spain	1	2015	42.9	-4.6
Mcrypticus_SA09	Spain	1	2014	42.0	-6.7
Mcrypticus_SA13	Spain	1	2014	43.0	-5.1
Mcrypticus_SA14	Spain	1	2014	42.9	-4.6
Mcrypticus_SA23	Spain	1	2014	42.9	-4.5
Mcrypticus_SA25	Spain	1	2014	42.9	-4.3
Mcrypticus_SA26	Spain	1	2014	41.9	-2.6
Mcrypticus_SA27	Spain	1	2014	42.0	-2.8
Mcrypticus_SA29	Spain	1	2012	42.6	0.5
Mcrypticus_SA30	Spain	1	2012	42.5	0.3
Mcrypticus_SA33	Spain	1	2012	42.6	-7.1
Mcrypticus_SA44	Spain	1	2013	42.0	-7.1
Mcrypticus_SA45	Spain	1	2011	42.7	0.6
Mcrypticus_SA46	Spain	1	2011	42.6	0.3

Mcrypticus_SA47	Spain	2	2011	42.2	-2.8
Mcrypticus_SA62	Spain	1	2011	42.6	0.7
Mcrypticus_SB65	Spain	1	2015	43.4	-7.8
Mcrypticus_SB68	Spain	1	2015	43.3	-6.5
Mcrypticus_SB71	Spain	1	2015	43.0	-4.9

Table S9. Landscape Resistance variables included in the Landscape Genetics analysis (Hansen et al. 2013: https://earthenginepartners.appspot.com/science-2013-global-forest/download_v1.5.html; GlobCover2009: http://due.esrin.esa.int/page_globcover.php; SRTM: https://www2.jpl.nasa.gov/srtm/; WorldClim: http://worldclim.org/version2).

Variable	Explanation	Resistance costs	Source map
ENM	Habitat suitability based on present ENMs with climatic and land cover variables	1-100 (highest-lowest suitability)	Generated with Maxent v3.4
tree cover	Percent tree cover	1-100 (100-0% tree cover)	Hansen et al. (2013)
broadleaf	Distance to broadleaf forests	1-100 (nearest-furthest)	GlobCover2009 map
conifer	Distance to conifer forests	1-100 (nearest-furthest)	GlobCover2009
forest	Distance to all forest types	1-100 (nearest-furthest)	GlobCover2009 map
lcfor50	Reclassified land cover map into forest vs non-forest land covers	1=forest; 50=other	GlobCover2009 map
lcfor75	Reclassified land cover map into forest vs non-forest land covers	1=forest; 75=other	GlobCover2009 map
lcfor100	Reclassified land cover map into forest vs non-forest land covers	1=forest; 100=other	GlobCover2009 map
landcov1	Reclassified land cover map into different costs to key land cover types	1=conifer/broadleaf; 5=broadleaf/conifer; 10=mosaic forest; 20=mosaic; 40=shrub & grass; 50=crops & water; 60=bare; 75=urban	GlobCover2009 map
landcov2	Reclassified land cover map into different costs to key land cover types	1=conifer/broadleaf; 5=broadleaf/conifer; 10=mosaic forest; 20=mosaic; 50=other land cover	GlobCover2009 map
landcov3	Reclassified land cover map into different costs to key land cover types	1=conifer/broadleaf; 5=broadleaf/conifer; 10=mosaic forest; 20=mosaic; 100=other land cover	GlobCover2009 map
altitude	Altitude maps	1-100 (lowest-highest)	SRTM map
slope	Slope calculated from Altitude map in ArcGIS 10.3	1-100 (gradual-steepest slope)	SRTM map
rugged	Topographic ruggedness calculated from Altitude map in ArcGIS 10.3	1-100 (lowest-highest level of ruggedness	SRTM map
autumn	September-November average temperatures	1-100 (highest-lowest temperatures)	WorldClim
rain3	September-November total rainfall	1=low <50mm; 50=medium 50-100mm; 100=high >100mm	WorldClim
rain6	September-November total rainfall	1=<50mm; 10=50-75mm; 25=75-100mm; 50=100-125mm; 75=125-150mm; 100=>150mm	WorldClim

Table S10. Genetic differentiation (Fst) between *M. escalerai* populations based on the neutral (top triangle; excluding Bayescan outliers and GEA SNPs) and full (bottom triangle) datasets.

	**Ast**	**Gir**	**Fra**	**Lit**	**Alg**	**Min**	**Bai**	**Zar**	**Gal**	**Cat**	**Gra**	**Sev**	**Seg**	**Pal**	**Cac**	**Hue**	**Cas**	**Val**
**Asturia**		0.295	0.321	0.078	0.120	0.068	0.072	0.171	0.080	0.187	0.116	0.102	0.141	0.132	0.080	0.193	0.166	0.145
**Girona**	0.335		0.063	0.255	0.292	0.246	0.247	0.166	0.285	0.147	0.208	0.236	0.176	0.192	0.238	0.156	0.156	0.166
**France**	0.359	0.066		0.280	0.317	0.270	0.273	0.199	0.310	0.180	0.236	0.263	0.206	0.222	0.264	0.190	0.190	0.196
**Litoral**	0.082	0.294	0.317		0.058	0.011	0.010	0.127	0.044	0.146	0.067	0.045	0.094	0.090	0.022	0.153	0.120	0.099
**Algarve**	0.127	0.332	0.354	0.059		0.048	0.058	0.165	0.092	0.182	0.096	0.071	0.133	0.133	0.065	0.188	0.158	0.134
**Minho**	0.071	0.284	0.305	0.012	0.049		0.004	0.118	0.028	0.136	0.060	0.041	0.085	0.079	0.015	0.142	0.110	0.091
**Baixa**	0.076	0.286	0.309	0.011	0.060	0.005		0.117	0.037	0.134	0.062	0.044	0.086	0.079	0.017	0.141	0.109	0.092
**Zaragoza**	0.202	0.171	0.204	0.155	0.195	0.144	0.146		0.146	0.036	0.075	0.106	0.037	0.047	0.100	0.038	0.021	0.037
**Galicia**	0.085	0.326	0.350	0.043	0.094	0.029	0.038	0.177		0.165	0.091	0.072	0.113	0.109	0.044	0.173	0.143	0.120
**Catalunya**	0.222	0.153	0.185	0.179	0.217	0.169	0.169	0.035	0.201		0.087	0.119	0.050	0.059	0.119	0.022	0.021	0.041
**Granada**	0.130	0.227	0.253	0.079	0.109	0.071	0.074	0.083	0.104	0.099		0.033	0.063	0.066	0.054	0.094	0.060	0.040
**Sevilla**	0.109	0.267	0.292	0.048	0.075	0.044	0.048	0.126	0.077	0.145	0.040		0.083	0.082	0.037	0.125	0.093	0.073
**Segovia**	0.161	0.186	0.215	0.114	0.154	0.104	0.106	0.038	0.135	0.055	0.069	0.097		0.037	0.072	0.056	0.033	0.039
**Palencia**	0.154	0.206	0.236	0.109	0.152	0.097	0.100	0.049	0.130	0.067	0.071	0.094	0.041		0.067	0.065	0.039	0.045
**Caceres**	0.086	0.271	0.295	0.023	0.068	0.019	0.020	0.121	0.048	0.146	0.061	0.040	0.086	0.081		0.125	0.091	0.077
**Huesca**	0.230	0.163	0.197	0.185	0.224	0.174	0.175	0.038	0.210	0.021	0.106	0.151	0.061	0.071	0.152		0.029	0.045
**Castellón**	0.199	0.163	0.197	0.149	0.189	0.138	0.140	0.021	0.174	0.021	0.069	0.115	0.037	0.045	0.115	0.029		0.019
**Valencia**	0.171	0.174	0.204	0.123	0.159	0.112	0.116	0.036	0.146	0.042	0.045	0.088	0.042	0.048	0.093	0.048	0.019	
